# Radiomics for personalised medicine: the long road ahead

**DOI:** 10.1038/s41416-019-0699-8

**Published:** 2020-01-15

**Authors:** Kenneth Miles

**Affiliations:** Institute of Nuclear Medicine, University College London, 5th Floor, Tower, University College Hospital, 235 Euston Road, London, NW1 2BU UK

**Keywords:** Cancer imaging, Tumour biomarkers

## Abstract

Radiomics is well placed to make clinically effective and cost-effective contributions to cancer care as a decision-making tool for personalised medicine. However, a systematic evaluative framework needs to be established so that these benefits can be demonstrated with confidence.

## Main

Recent years have seen a major shift in medical imaging in which the observational and interpretative skills of the radiologist are increasingly supplemented by quantitative approaches to the characterisation of human tissues. Advances in information technology have allowed multiple imaging measurements to be derived from a single examination. These large arrays of imaging data can be analysed to enhance decision support for patients with cancer, a method now known as radiomics.

In this issue of the *British Journal of Cancer*, Wang and co-authors report a magnetic resonance imaging (MRI)-based radiomic model that provides prognostic information for patients with hepatocellular carcinoma undergoing curative hepatectomy.^1^ They propose that the risk stratification afforded by their technique can potentially identify subsets of patients who may benefit from the escalation of therapy and/or intensification of post-operative surveillance. Their work provides an excellent example of how medical imaging can potentially make a significant contribution to the era of personalised medicine.

A recent systematic review of the literature to 2018 has documented a rapid growth in radiomic research in recent years.^2^ The vast majority of studies come from the oncology field with a significant number reporting the ability of radiomics to determine prognosis or predict treatment response. This research heralds the future application of radiomics to personalised medicine for a wide range of cancer types. Before or during therapy, a radiomic model may indicate whether the treatment plan should be changed or escalated because the patient is predicted to gain insufficient benefit from current treatment. After treatment, a radiomic model may show whether a particular patient would benefit from more intense post-treatment surveillance due to a high risk of a tumour recurrence.

**Fig. 1:**
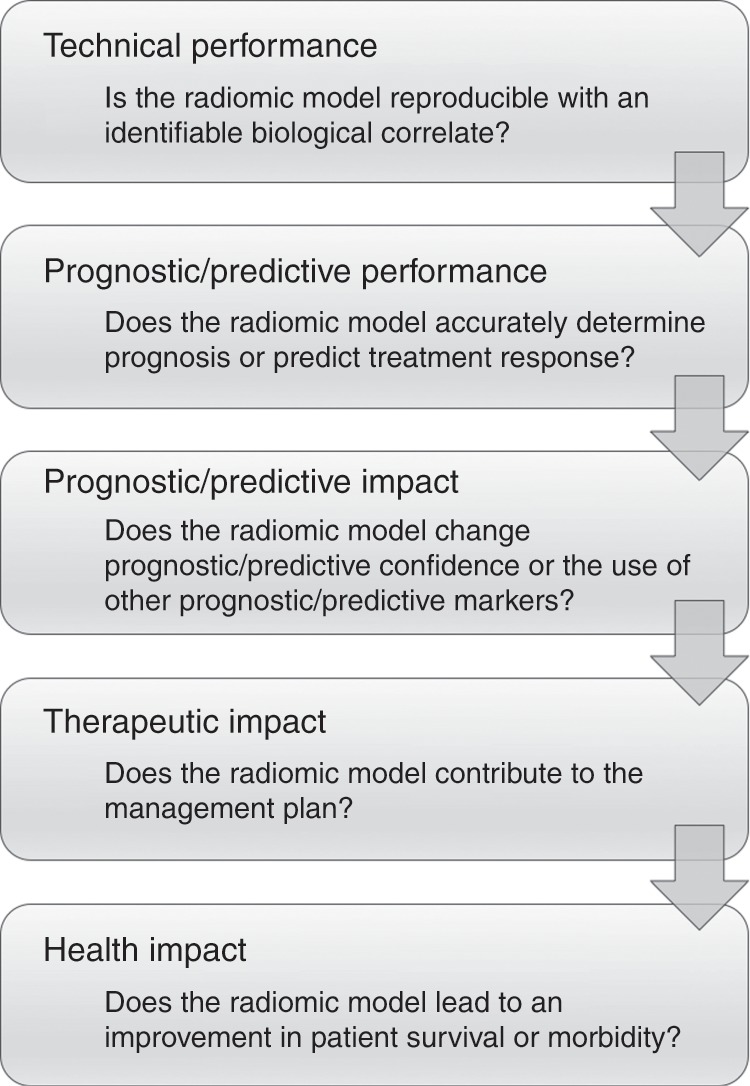
Flow chart outlining a hierarchical approach to the evaluation of radiomic models for prognostication and/or prediction of treatment response.

Before these scenarios can be realised in clinical practice, radiomic technologies require a systematic evaluation of their properties and effects as decision-making tools in healthcare. Although there is a well-established evaluative framework for diagnostic applications of medical imaging, a corresponding framework is yet to be established for radiomics as applied to personalised medicine. Nevertheless, the hierarchical approach used for diagnostic imaging^3^ can provide a basis for an equivalent framework for the evaluation of radiomics for prognostication or prediction of treatment response (Fig. 1). The most notable differences from the diagnostic approach lie in the assessment of technical and prognostic/predictive performance.

## Technical performance

The translation of radiomics into clinical practice is potentially rapid because the approach exploits imaging devices and techniques that are already approved for diagnostic use. Nevertheless, radiomic models need to be reproducible across different imaging systems, and for the same system over time. Many of the image-based measurements used in radiomic models are sensitive to changes in image acquisition and processing, which in turn may vary between imaging departments and equipment manufacturers. The technical quality assurance processes needed to control this variability are currently underdeveloped or non-existent. There is also a lack of commercially available software for the extraction, reporting, communicating and archiving of radiomic data. Regulatory approval of this software is potentially challenging, particularly if the radiomic model is combined with a deep-learning algorithm or entails a clinical role that is not currently part of medical imaging practice.

Clinicians may be reluctant to adopt radiomics if they cannot provide a rationale for any medical decision based on their use. This justification could be problematic if the biological significance of the image measurements underlying the radiomic model is unknown. Yet, there may be no direct one-to-one relationship between the phenotypic characteristics reflected by radiomic data and specific histopathological or genomic markers. The closest biological correlates may also have no demonstrable prognostic or predictive value. The more imaging features included in a radiomic model, the more problematic this issue becomes. Nevertheless, correlating radiomic features with genomic characteristics of known prognostic or predictive significance can accelerate the identification of radiomic models most likely to be clinically useful.^4^

## Prognostic/predictive performance

The methodologies required to show that a radiomic model accurately determines prognosis or predicts treatment response are only beginning to be established within the imaging community. Effective evaluation criteria and reporting guidelines are needed to minimise bias and reduce the risk of overly optimistic conclusions. The effect size should be greater than established clinical or pathological markers of prognosis or treatment response, as well as simpler imaging measurements such as tumour size. A radiomics quality score has recently been proposed to aid the assessment of radiomic studies.^5^

### Beyond prognostic/predictive performance

Satisfactory technical, prognostic or predictive performance are necessary but not sufficient conditions for the clinical adoption of radiomics. It is also necessary to demonstrate the impact on prognostic/predictive confidence, therapeutic strategy and health outcomes. Although the methodologies for obtaining this evidence are broadly similar to those used for diagnostic imaging, these aspects have rarely been addressed in radiomic research to date.

The economic impact of radiomics is also an important consideration. By extracting additional information from medical images that are acquired as part of routine clinical care, radiomics can potentially achieve health impact at relatively low cost. Reductions in health expenditure might be achievable from fewer patients undergoing surveillance (by targeting post-treatment surveillance to high-risk patients) and savings from avoidance or withdrawal of unbeneficial treatment (through the identification of patients unlikely to receive sufficient benefit from treatment). A formal economic evaluation of radiomics is yet to be performed.

## Conclusions

At the end of the 20th century, the dominant paradigm in the clinical evaluation of emerging imaging technologies was determination of diagnostic accuracy. Today, the status of radiomic research is comparable with the dominant paradigm being evaluation of prognostic or predictive performance. Although current studies point to a future role for radiomics as a decision-making tool in clinical oncology, there is much to be done before the evidence of health impact and cost-effectiveness is available to justify the clinical use of radiomics for personalised medicine.

## Data Availability

Not applicable.
